# Whole-Genome Sequencing Provides Insight Into Antimicrobial Resistance and Molecular Characteristics of *Salmonella* From Livestock Meat and Diarrhea Patient in Hanzhong, China

**DOI:** 10.3389/fmicb.2022.899024

**Published:** 2022-06-09

**Authors:** Rui Weng, Yihai Gu, Wei Zhang, Xuan Hou, Hui Wang, Junqi Tao, Minghui Deng, Mengrong Zhou, Yifei Zhao

**Affiliations:** ^1^Department of Microbiology, 3201 Hospital, School of Medicine, Xi’an Jiaotong University, Hanzhong, China; ^2^Department of Medical Technology, Shaanxi University of Chinese Medicine, Xianyang, China; ^3^The Second Clinical Medical College of Nanchang University, Nanchang, China

**Keywords:** *Salmonella*, antimicrobial resistance, whole-genome sequencing, livestock meat, diarrhea patients

## Abstract

*Salmonella* is a major zoonotic pathogen, which usually contaminates food resulting in salmonellosis in humans. Exploring the characteristics and origins of *Salmonella* is essential in formulating prevention and control measures for *Salmonella* infection. We used slide agglutination, antimicrobial susceptibility testing, and whole-genome sequencing to analyze and compare *Salmonella*’s phenotype, genotyping diversity, and genetic relatedness from livestock meat and diarrhea patients in Hanzhong, China, from 2018 to 2020. Totally 216 *Salmonella* enterica isolates were screened from frozen whole chicken carcasses (44.3%, 70/158), frozen raw ground pork (36.2%, 59/163), and diarrhea patients (4.4%, 87/1964). *Salmonella* Typhimurium was the dominant serotype. Notably, compared with other sources, isolates obtained from frozen whole chicken carcasses showed significant resistance to third-generation cephalosporin and fluoroquinolones (*p* < 0.05). All strains were assigned into 36 sequence types (STs) and two novel STs, and an excellent consistency was observed between ST and serotype. Genomic data revealed that extended-spectrum β-lactamase genes were responsible for third-generation cephalosporin resistance in 52 *Salmonella* strains, and the most predominant resistance determinant was *bla*_CTX–M_. Furthermore, of the 60 ciprofloxacin-resistant isolates, five single-base mutations in quinolone resistance-determining regions were identified in *gyrA* or *parC*, and the plasmid-mediated quinolone resistance gene *aac(6’)Ib-cr* was most often detected. The cgMLST clusters show that five clusters among four serotypes (including *S*. Typhimurium, *S*. London, *S*. Derby, and *S*. Agona) cover samples from diarrhea patients and livestock meat pathway isolate, indicating a possibility of cross-host transmission. In conclusion, the livestock meat isolates have a higher level of resistance than diarrhea patients’ isolates and could be an essential source of human *Salmonella* infection.

## Introduction

Infections from foodborne pathogens have occurred worldwide and pose a significant threat to global public health. *Salmonella* is one of the four most crucial diarrhea diseases globally after *Campylobacter* (European Food Safety Authority, 2021a). In 2019, 87,923 cases of *Salmonella*, 16,628 hospitalizations, and 140 deaths were reported in the European Union (EU) (European Food Safety Authority, 2021a). In China, 70–80% of bacterial food poisoning is caused by *Salmonella*. In addition, there are about 9.87 million cases of gastroenteritis caused by non-typhoid *Salmonella* each year (Techniques et al., 2011).

*Salmonella* is divided into two species: *Salmonella* bongori and *Salmonella* enterica (*S*. enterica), with the latter comprising six different *subspecies*, and currently, more than 2,600 serotypes have been recognized worldwide (Monte et al., 2021). About 99% of *Salmonella* strains that result in infections in humans or mammals are *S*. enterica (Kurtz et al., 2017). In 2019, the top five serotypes reported in the EU associated with human infection were distributed: *S*. Infantis, *S*. Enteritidis, monophasic variant of *S*. Typhimurium, *S*. Typhimurium, and *S*. Derby (European Food Safety Authority, 2021a). In China, *S*. Enteritidis and *S*. Typhimurium are the most common serotypes of human intestinal infections (Havelaar et al., 2015; Wang et al., 2020).

The phenotyping and genotyping of *Salmonella* are diverse. In a recent study showing 250 *S*. enterica were isolated from different sources covering nine provinces of China from 2004 to 2019, all strains were assigned into 36 serovars and 43 sequence types (STs) (Yan et al., 2021). Antibiotics are used to treat severe *Salmonella* infections. At present, quinolones and third-generation cephalosporin are essential antimicrobials for human salmonellosis (Zhan et al., 2017). Another study showed that a total of 218 *Salmonella* were collected from retail meat, covering most provincial capitals in China. The resistance rates of ciprofloxacin (CIP), ceftriaxone, and cefotaxime (CTX) were 16.1%, 5.5%, and 4.6%, respectively (Yang et al., 2019). In 2019, the EU reported data on antimicrobial resistance in *Salmonella* isolates from human cases of non-typhoidal salmonellosis. The highest rate of resistance of human *Salmonella* isolates was observed for sulfonamides (SUL, 29.0%), ampicillin (AMP, 25.8%), and tetracyclines (TET, 25.6%). Furthermore, the proportion of *Salmonella* isolates resistant to the critically important antimicrobial CIP, CTX, and ceftazidime (CAZ) was 13.5%, 1.8%, and 1.2%, respectively (European Food Safety Authority, 2021b). Thus, the resistance of those highest priority “critically important antimicrobials” requires great attention.

Evidence implies an association between the use of antimicrobials in food animals and resistant bacteria isolated from humans (Swartz, 2002). Approximately 80% of foodborne animals are currently being treated with veterinary drugs for some or all of their life (Pavlov et al., 2008). Antibiotic usage in the livestock industry may leave antibiotic residues in foods, such as meat, egg, and milk (Bacanlı and Başaran, 2019). Live poultry and poultry meat are regarded as the primary vehicles for human salmonellosis (Foley et al., 2008; Majowicz et al., 2010). Accordingly, there has a higher health risk of salmonellosis. With the increasing globalization of livestock meat and other foods, the resistance monitoring and infection control of *Salmonella* are strongly necessary.

This study aimed to explore the phenotyping and genotyping diversities and genetic relationships of *Salmonella* from different sources, including frozen whole chicken carcasses, frozen raw ground pork, and diarrhea patients, based on the slide agglutination, antimicrobial susceptibility testing, and whole-genome analyses.

## Materials and Methods

### Sample Collection and Identification

From January 2018 to August 2020, 321 livestock meat samples were collected from seven different large supermarkets. Samples included frozen whole chicken carcasses (*n* = 158) and frozen raw ground pork (*n* = 163) (the weight of each sample was greater than 200 g). The human origin samples (*n* = 1964) were from feces of diarrhea patients from the 3201 hospital, Hanzhong, Shannxi, China, one of the largest medical institutions visited most frequently by the patients in the area. All samples from different sources were collected from Hanzhong City, China.

All livestock meat samples were transported to the laboratory within 1 h, each frozen whole chicken carcasses and 100 g of frozen raw ground pork were immediately placed in sterile homogenizer bags, then 500 mL and 800 mL buffered peptone water (BPW; LAND BRIDGE, Beijing, China) were added, respectively. The bag was manually massaged for 3–5 min and kept for overnight incubation at 37 ± 1°C. Next, 100 μL whole chicken carcass or raw ground pork rinse samples were transferred to 9 mL Rappaport-Vassiliadis (RV; LAND BRIDGE, Beijing, China) broth and incubated at 42 ± 1°C for 18 h. After enrichment, a loopful of RV broth culture was streaked on Xylose lysine Desoxycholate agar (XLD; Dijing, Guangzhou, China) and incubated at 37 ± 1°C overnight. Suspected colonies were identified with matrix-assisted laser desorption/ionization time-of-flight mass spectroscopy (MALDI-TOF MS; Bruker, Bremen, Germany). Similarly, fecal samples were cultured on XLD and confirmed with MALDI-TOF MS. After isolation, strains were stored at −80°C in Luria-Bertani (LB) broth with 50% glycerol.

### *Salmonella* Serotyping *via* Slide Agglutination

Confirmed *Salmonella* isolates were serotyped based on the Kauffmann–White scheme (Grimont and Weill, 2007) and National Food Safety Standard food microbiological examination: *Salmonella* (GB 4789.4-2016) (Ministry of Health of the People’s Republic of China, 2016) by slide agglutination with commercial *Salmonella* antisera (Statens Serum Institute, Denmark).

### Antimicrobial Susceptibility Testing

Antimicrobial susceptibility testing of all *Salmonella* isolates was performed using the broth microdilution method according to the manufacturer’s instructions (Xingbai, Shanghai, China). Susceptibility to the following 25 antimicrobials was tested: AMP, cefazolin (CFZ), cefoxitin, CTX, CAZ, cefepime (FEP), meropenem (MEM), imipenem (IMP), CIP, levofloxacin (LEV), nalidixic acid (NAL), azithromycin (AZI), colistin (CT), polymyxin B (PB), TET, SUL, amikacin (AMI), kanamycin (KAN), gentamicin (GEN), streptomycin (STR), chloramphenicol (CHL), aztreonam (AZM), amoxicillin-clavulanic acid, ampicillin-sulbactam, and trimethoprim-sulfamethoxazole. Escherichia *coli* ATCC 25922 was used as a quality control strain. All susceptibility results were interpreted according to the Clinical and Laboratory Standards Institute (CLSI, 2020) interpretive standards (CLSI, 2020) and European Committee on Antimicrobial Susceptibility (CLSI,2020) Testing (EUCAST) breakpoint interpretation (EUCAST, 2021). Extended-spectrum β-lactamase (ESBL) production was determined by *a* ≥ 3 2-fold concentration decrease in any minimum inhibitory concentration (MIC) of CTX or CAZ combined with clavulanic acid versus its MIC when tested alone (CLSI, 2005).

### Whole Genome Sequencing

Genomic DNA was extracted from all *Salmonella* isolates by QIAamp DNA Mini Kit (QIAGEN, Germany). The Illumina HiSeq X-Ten platform (Illumina, United States) with 2 bp × 150 bp ends was used to generate genome sequence raw data which were subsequently assembled by the Shovill (0.9.0). Oxford Nanopore MinION platforms were applied to sequence the carbapenem-resistance strain whole genomic DNA and hybrid long read-short read assemblies were conducted using Unicycler (0.4.8).

The Multi Locus Sequence Typing (MLST 2.0) and antimicrobial resistance genes (ResFinder 4.1) were performed by the tool of the Center for Genomic Epidemiology (CGE)^1^. *Salmonella in silico* Typing Resource (SISTR)^2^ was evaluated for *in silico* determination of *Salmonella* serotypes using whole genome sequencing (WGS) data. Virulence factors were analyzed using the Virulence Factor Database (VFDB)^3^. Ridom SeqSphere + 4.1.9 software (Ridom, Munster, Germany) was used to perform MLST and core-genome multilocus sequence typing (cgMLST) analysis and visualized in the minimum spanning trees. A cluster was defined as a group of closely related MLST-analyzed and cgMLST-analyzed isolates with a single-linkage threshold of ≤ 1 and 7 alleles, respectively (Dangel et al., 2019).

### Statistical Analysis

The Chi-squared test and Fisher exact probability test were performed using SPSS (IBM version 26.0). The Bonferroni-corrected *P*-values that are less than 0.05 were considered to indicate statistical significance.

## Results

### Prevalence and Serotype of *Salmonella* in Different Sources

We collected 216 *Salmonella* strains, among which 40.2% (129/321) were isolated from livestock meat samples and 4.4% (87/1964) were from diarrhea patients. In the poultry meat samples, the isolation rate in frozen whole chicken carcasses (44.3%, 70/158) was relatively higher than that in frozen raw ground pork (36.2%, 59/163). Correspondingly, the isolation rate in diarrhea patients was significantly lower (*P* < 0.05). Thirty-four serotypes of *Salmonella* were identified *via* slide agglutination, except for three untypeable isolates (Table 1). From 70 whole chicken carcasses isolates, 13/70 (18.6%) were identified as *S*. Kentucky, followed by *S*. Agona (9/70, 12.9%), *S*. Enteritidis (5/70, 7.1%), *S*. Derby (5/70, 7.1%), and *S*. Schwarzengrund (5/70, 7.1%), which occupied the top five serotypes. Of the 59 raw ground pork isolates, the most common serotypes were *S*. Typhimurium and its monophasic variant (33.9%, 20/59), *S*. Derby (25.4%, 15/59), and *S*. Rissen (18.6%, 11/59). Among the 87 human fecal samples, *S*. Typhimurium and its monophasic variant (46.0%, 40/87) and *S*. Enteritidis (25.3%, 22/87) were the dominant serotypes. The overlap and diversity of dominant serotypes occurred between the sources described earlier.

**TABLE 1:** Serotypes *via* slide agglutination and the corresponding number of *Salmonella* isolates.

Serotype	Source of isolate			Total (*n* = 216)
	
	Frozen whole chicken carcasses (*n* = 70)	Frozen raw ground pork (*n* = 59)	Human (*n* = 87)	
*S*. Typhimuium	2	20	40	62
*S*. Enteritidis	5	0	22	27
*S*. Derby	4	15	4	23
*S*. Rissen	2	11	1	14
*S*. Kentucky	13	0	1	14
*S*. Agona	9	2	2	13
*S*. London	1	4	5	10
*S*. Indiana	5	0	1	6
*S*. Schwarzengrund	5	1	0	6
*S*. Give	1	3	0	4
*S*. Newport	0	0	3	3
*S*. kottbus	3	0	0	3
*S*. Havana	3	0	0	3
*S*. Saintpaul	0	0	2	2
*S*. Stanley	0	0	2	2
S. Goldcoast	1	0	1	2
*S*. Thompson	1	0	1	2
*S*. I 4,[5],12:b:-	0	0	1	1
*S*. Weltevreden	0	0	1	1
*S*. Reading	0	1	0	1
*S*. Ruzizi	0	1	0	1
*S*. Muenster	0	1	0	1
*S*. Albany	1	0	0	1
*S*. Kedougou	1	0	0	1
*S*. Corvallis	1	0	0	1
*S*. Larochelle	1	0	0	1
*S*. Rechovot	1	0	0	1
*S*. Mbandaka	1	0	0	1
*S*. Magherafelt	1	0	0	1
*S*. Chomedey	1	0	0	1
*S*. Worthington	1	0	0	1
*S*. Singapore	1	0	0	1
*S*. Idikan	1	0	0	1
*S*. Bonmriensis	1	0	0	1
*Salmonella* app.	3	0	0	3

### Antimicrobial Susceptibility Phenotypes

Out of 216 *Salmonella* isolates, only 3 (1.4%, 3/216) were susceptible to all antimicrobial agents tested (Table 2), and the streptomycin (STR) resistance rate was highest (80.6%, 174/216), followed by TET and AMP (76.9%, 166/216). A total of 47 (21.8%, 47/216) ESBLs-producing *Salmonella* were identified. Notably, one (0.5%, 1/216) isolate from frozen whole chicken carcasses was resistant to carbapenems. The MIC values of MEM and IMP were 64 μg/mL and 32 μg/mL, respectively. In addition, the isolates cultured from whole chicken carcasses showed significantly higher resistance to CFZ (65.7%), CTX (54.3%), CAZ (41.4%), FEP (52.9%), CIP (54.3%), LEV (51.4%), NAL (60.0%), AZI (37.1%), AMI (17.1%), KAN (52.9%), GEN (50.0%), and AZM (52.9%) than those from frozen raw ground pork and/or diarrhea patients (*p* < 0.05). The resistance of isolates obtained from raw ground pork to TET (93.2%) was significantly higher than that of diarrhea patients (*p* < 0.05). Moreover, the isolates cultured from diarrhea patients showed significantly higher resistance to CT (31.0%) and PB (24.1%) than those from the other samples (*p* < 0.05). It was observed that the *Salmonella* obtained from livestock meat showed higher resistance to essential drugs compared with isolates cultured from patients with diarrhea, especially strains isolated from whole chicken carcasses.

**TABLE 2:** Antimicrobial susceptibility testing and comparison of the resistance of *Salmonella* isolates from different sources.

Antimicrobial Agent	Number of isolates (%)				χ2/P							
		
	FWCC (*n* = 70)	FGRP (*n* = 59)	H (*n* = 87)	Total (*n* = 216)	All sources		FWCC vs. FGRP		FWCC vs. H		FGRP vs. H	
AMP	52 (74.29)	49 (83.05)	65 (74.71)	166 (76.85)	1.7570	0.4150	/	/	/	/	/	/
AMC	7 (10.00)	4 (6.78)	13 (14.94)	24 (11.11)	2.5010	0.2860	/	/	/	/	/	/
AMS	45 (64.29)	38 (64.41)	52 (59.77)	135 (62.50)	0.4630	0.7930	/	/	/	/	/	/
CFZ	46 (65.71)	20 (33.90)	41 (47.13)	107 (49.54)	13.3030	**0.0010**	12.9700	**0.0000**	5.4240	0.0200	2.5290	0.1120
CFX	1 (1.43)	0 (0.00)	4 (4.60)	5 (2.31)	/	0.2250*	/	/	/	/	/	/
CTX	38 (54.29)	3 (5.08)	11 (12.64)	52 (24.07)	52.8130	**0.0000**	37.7450	**0.0000**	31.3300	**0.0000**	2.3170	0.1280
CAZ	29 (41.43)	2 (3.39)	5 (5.75)	36 (16.67)	45.8600	**0.0000**	25.3750	**0.0000**	29.1080	**0.0000**	/	0.7020*
FEP	37 (52.86)	3 (5.08)	6 (6.90)	46 (21.30)	61.6140	**0.0000**	34.1540	**0.0000**	41.2020	**0.0000**	/	0.7400*
MEM	1 (1.43)	0 (0.00)	0 (0.00)	1 (0.46)	/	0.5970*	/	/	/	/	/	/
IMP	1 (1.43)	0 (0.00)	0 (0.00)	1 (0.46)	/	0.5970*	/	/	/	/	/	/
CIP	38 (54.29)	10 (16.95)	12 (13.79)	60 (27.78)	36.4470	**0.0000**	19.1020	**0.0000**	29.3030	**0.0000**	0.2740	0.6010
LEV	36 (51.43)	5 (8.47)	2 (2.30)	43 (19.91)	65.3760	**0.0000**	27.2450	**0.0000**	51.0360	**0.0000**	/	0.1190*
NAL	42 (60.00)	13 (22.03)	35 (40.23)	90 (41.67)	18.9140	**0.0000**	18.8680	**0.0000**	6.0670	**0.0140**	5.2750	0.0220
AZI	26 (37.14)	4 (6.78)	5 (5.75)	35 (16.20)	33.4690	**0.0000**	16.5380	**0.0000**	24.1280	**0.0000**	/	1.0000*
CT	6 (8.57)	1 (1.96)	27 (31.03)	34 (15.74)	26.8320	**0.0000**	/	0.1240*	11.7900	**0.0010**	19.5250	**0.0000**
PB	4 (5.71)	0 (0.00)	21 (24.14)	25 (11.57)	23.4890	**0.0000**	/	0.1250*	9.8350	**0.0020**	16.6340	**0.0000**
TET	56 (80.00)	55 (93.22)	55 (63.22)	166 (76.85)	18.3660	**0.0000**	4.6600	0.0310	5.2740	0.0220	17.0340	**0.0000**
SUL	35 (50.00)	28 (47.46)	54 (62.07)	117 (54.17)	3.7480	0.1540	/	/	/	/	/	/
SXT	42 (60.00)	35 (59.32)	23 (26.44)	100 (46.30)	23.1140	**0.0000**	0.0600	0.9380	18.0110	**0.0000**	15.8790	**0.0000**
AMI	12 (17.14)	0 (0.00)	0 (0.00)	12 (5.56)	/	0.0000*	11.1520	**0.0010**	11.1520	**0.0010**	/	/
KAN	37 (52.86)	10 (16.95)	8 (9.20)	55 (25.46)	42.0620	**0.0000**	17.8240	**0.0000**	36.1650	**0.0000**	1.9560	0.1620
GEN	35 (50.00)	13 (22.03)	8 (9.20)	56 (25.93)	34.2710	**0.0000**	10.7170	**0.0010**	32.4760	**0.0000**	4.7060	0.0300
STR	57 (81.43)	48 (81.36)	69 (79.31)	174 (80.56)	0.1440	0.9300	/	/	/	/	/	/
CHL	41 (58.57)	33 (55.93)	30 (34.48)	104 (48.15)	10.9860	**0.0400**	0.0910	0.7630	9.8060	**0.0030**	6.5940	**0.0100**
AZM	37 (52.86)	5 (8.47)	8 (9.20)	50 (23.15)	51.3910	**0.0000**	28.7210	**0.0000**	36.1650	**0.0000**	0.0230	0.8810

*AMP, ampicillin; AMC, amoxicillin-clavulanic acid; AMS, ampicillin-sulbactam; CFZ, cefazolin; CFX, cefoxitin; CTX, cefotaxime; CAZ, ceftazidime; FEP, cefepime; MEM, meropenem; IMP, imipenem; CIP, ciprofloxacin; LEV, levofloxacin; NAL, nalidixic acid; AZI, azithromycin; CT, colistin; PB, polymyxin B; DOX, doxycycline; TET, tetracycline; SUL, sulfisoxazole; SXT, trimethoprim-sulfamethoxazole; AMI, amikacin; KAN, kanamycin; GEN, gentamicin; STR, streptomycin; CHL, chloramphenicol; AZM, aztreonam; H, human; FWCC, frozen whole chicken carcasses; FGRP, frozen raw ground pork.*

The “/” means that the data cannot be calculated, the “*” means that the data were calculated with Fisher’s exact test.

*The bold shows that there is a significant difference between different sources.*

**FIGURE 1:**
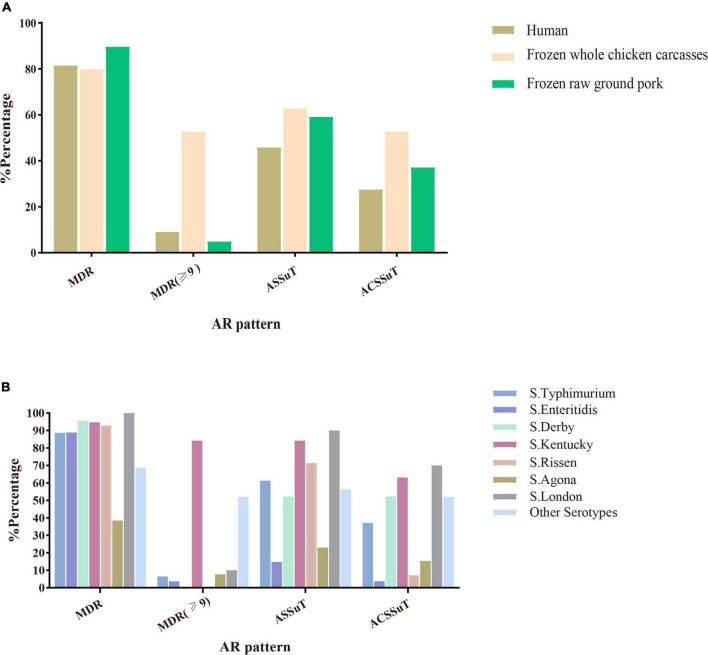
Antimicrobial susceptibility patterns (MDR, ASSuT, and ACSSuT) in 216 *Salmonella* isolates from different sources **(A)** and serotypes **(B)**. MDR, multidrug-resistant; MDR (≥ 9): ≥ 9 antimicrobial classes; ASSuT, ampicillin, streptomycin, sulfonamides, and tetracycline; ACSSuT, ampicillin, chloramphenicol, streptomycin, sulfonamides, and tetracycline.

Among the 216 *Salmonella* isolates, 180 (83.3%, 180/216) were defined as multidrug-resistant (MDR) isolates resistant to three or more antimicrobial classes. Of these, even 48 (26.7%, 46/180) were resistant to nine or more antimicrobial classes we tested. *S*. Kentucky (34.8%, 16/46) cultured from frozen whole chicken carcasses occupied the largest proportion of this. Besides this, two important resistance patterns (Wang et al., 2019) were also analyzed in this study: 55.1% (119/216) of 216 *Salmonella* isolates showed ASSuT (AMP, STR, SUL, and TET) resistance pattern and 38.4% (83/216) of isolates showed ACSSuT (AMP, CHL, STR, SUL, and TET) resistance pattern. Figure 1 showed the antibiotic resistance patterns in *Salmonella* isolates obtained from different sources (Figure 1A) and serotypes (Figure 1B). Obviously, compared to isolates from the other two sources, isolates from whole chicken carcasses were more resistant to antibacterial agents. Moreover, *S*. Kentucky was resistant to more antimicrobial classes than other serotypes.

### *In silico* Serotyping Based on Whole-Genome Sequencing

*In silico* serotype prediction based on the genome sequence of 216 *Salmonella* isolates showed that the strains had 31 different serotypes (Table 3). The concordance rate between *in silico* serotype prediction and traditional serology was 94.0% (203/216). Except for the three isolates identified as *Salmonella* spp. by slide agglutination, the serogroup of other isolates using the two methods was consistent. The discordant strains were identified *in silico* as *S*. Molade, *S*. London, *S*. Kentucky, *S*. Thompson, *S*. Lille, *S*. Give, *S*. Senftenberg, *S*. Ouakam, and *S*. E1:l,v:l,z13,z28, according to these samples order rather than *S*. Chomedey, *S*. Give, *S*. Corvallis, *S*. Rechovot, *S*. London, *S*. Bonmriensis, *S*. Magherafelt, *S*. Singapore, *S*. Larochelle, and *S*. Ruzizi by traditional typing method, respectively. In this study, 15 serotypes were identified in fecal samples from patients with diarrhea, and 26 serotypes were identified in livestock meat samples. It appears that the serotype distribution of *Salmonella* isolated among livestock meat was more diverse. Interestingly, specific serotype appears to be related to a particular origin, some serotypes, including *S*. Newport, *S*. Saintpaul, *S*. Stanley, Paratyphi B var. Java monophasic, and *S*. Weltevreden, were exclusively detected among fecal samples. On the contrary, *S*. Schwarzengrund, *S*. Give, *S*. Havana, *S*. Kottbus, *S*. Albany, *S*. E1:l,v:l,z13, z28, *S*. Idikan, *S*. Kedougou, *S*. Lille, *S*. Mbandaka, *S*. Molade, *S*. Muenster, *S*. Ouakam, *S*. Reading, *S*. Senftenberg, and *S*. Worthington were only found among livestock meat samples.

**TABLE 3:** *In silico* serotype and the corresponding number of *Salmonella* isolates.

*In silico* serotype	Source of isolate			Total (*n* = 216)
	
	Frozen whole chicken carcasses (*n* = 70)	Frozen raw ground pork (*n* = 59)	Human (*n* = 87)	
*S*. Typhimurium	2	20	40	62
*S*. Enteritidis	5	0	22	27
*S*. Derby	4	15	4	23
*S*. Kentucky	18	0	1	19
*S*. Rissen	2	11	1	14
*S*. Agona	9	2	2	13
*S*. London	0	5	5	10
*S*. Indiana	5	0	1	6
*S*. Schwarzengrund	5	1	0	6
*S*. Give	2	2	0	4
*S*. Havana	3	0	0	3
*S*. Kottbus	3	0	0	3
*S*. Newport	0	0	3	3
*S*. Thompson	2	0	1	3
*S*. Goldcoast	1	0	1	2
*S*. Saintpaul	0	0	2	2
*S*. Stanley	0	0	2	2
*S*. Albany	1	0	0	1
*S*. E1:l,v:l,z13,z28	0	1	0	1
Paratyphi B var. Java monophasic	0	0	1	1
*S*. Idikan	1	0	0	1
*S*. Kedougou	1	0	0	1
*S*. Lille	1	0	0	1
*S*. Mbandaka	1	0	0	1
*S*. Molade	1	0	0	1
*S*. Muenster	0	1	0	1
*S*. Ouakam	1	0	0	1
*S*. Reading	0	1	0	1
*S*. Senftenberg	1	0	0	1
*S*. Weltevreden	0	0	1	1
*S*. Worthington	1	0	0	1

### Molecular Typing and Genetic Relatedness Analysis

**FIGURE 2:**
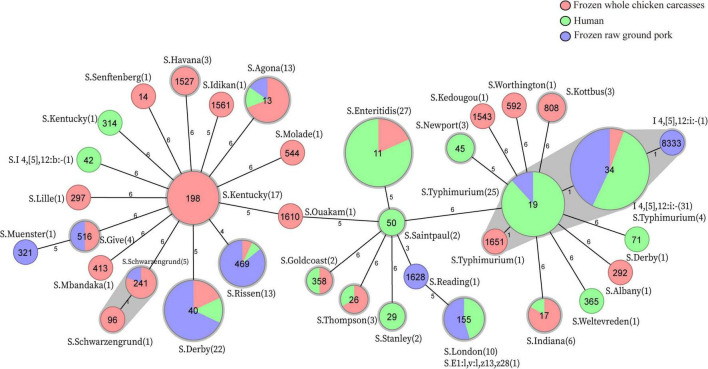
MLST-based Minimum Spanning Tree and *in silico* serotype distribution of 214 *Salmonella*, two strains S-3-16 and S-5-8 were excluded due to the presence of the new allele of housekeeping gene *hisD* and *thrA*, respectively. The sequence type is represented in the circle. Numbers in brackets indicate the number of strains corresponding to the *in silico* serotype.

**FIGURE 3:**
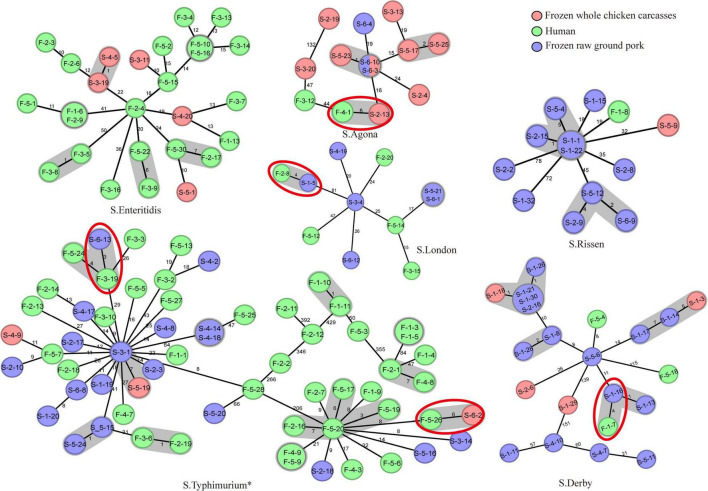
cgMLST-based Minimum Spanning Tree of the six serotypes (*S*. Enteritidis, *S*. Typhimurium, *S*. London, *S*. Derby, *S*. Rissen, and *S*. Agona) that included both diarrhea patients and poultry meat pathway isolates. The red-dotted circles indicate the cluster covering samples from diarrhea patients and frozen whole chicken carcasses or raw ground pork. The strain name is represented in the circle. The “*” means *S*. Typhimurium and its monophasic variant.

In total, MLST analysis revealed 36 STs and two novel STs (Table 4) among 216 *Salmonella* isolates. ST34 (16.2%, 35/216), ST11 (12.5%, 27/216), and ST19 (11.6%, 25/216) were the three most common prevalent STs. A comparison of MLST and serotyping showed that each serotype comprised one ST and vice versa. Sixty-two isolates with *S*. Typhimurium or its monophasic variant correspond to four STs, including ST34 (56.5%, 35/62), ST19 (40.3%, 25/62), and so forth. ST40 (95.7%, 22/23) and ST71 (4.3%, 1/23) were identified in *S*. Derby. ST469 (92.9%, 13/14) and one novel ST were identified in *S*. Rissen. Beyond this, 19 *S*. Kentucky belong to ST198 (89.5%, 17/19), ST314 (5.3%, 1/19), and one novel ST. Thus, the distinguishability of bacterial molecular typing using MLST seemed higher than that of serotyping. Nonetheless, there was an excellent consistency between ST typing and serotype, which was presented in Figure 2. Overall, serotyping is still serving as a gold-standard technique for routine typing in a common outbreak investigation. Moreover, the MLST cluster indicated that *S*. Typhimurium and its monophasic variant may be closely genetically related. In addition, five MLST clusters included both diarrhea patients and livestock meat pathway isolates, including *S*. Enteritidis, *S*. Typhimurium, and its monophasic variant, *S*. London, *S*. Derby, *S*. Rissen, and *S*. Agona. Therefore, we further identified cgMLST clusters within these five serotypes (Figure 3). The phylogenetic analysis based on core genomes showed that five clusters among four serotypes (including *S*. Typhimurium and its monophasic variant, *S*. London, *S*. Derby, and *S*. Agona) cover samples from different sources. Each of the five clusters included at least two genetically related samples with an internal core gene distance ranging from three to six alleles, implying a potential transmission of *Salmonella* happening between humans and domestic animals, even though the transmission route might be complicated and need to be further explored.

**TABLE 4:** Distribution of *Salmonella* isolates of Sequence Typing (ST).

ST	Source of isolate			Total (*n* = 216)
	
	Frozen whole chicken carcasses (*n* = 70)	Frozen raw ground pork (*n* = 59)	Human (*n* = 87)	
ST34	2	15	18	35
ST11	5	0	22	27
ST19	0	3	22	25
ST40	4	15	3	22
ST198	17	0	0	17
ST13	9	2	2	13
ST469	1	11	1	13
ST155	0	6	5	11
ST17	5	0	1	6
ST241	4	1	0	5
ST516	2	2	0	4
ST26	2	0	1	3
ST45	0	0	3	3
ST808	3	0	0	3
ST1527	3	0	0	3
ST29	0	0	2	2
ST50	0	0	2	2
ST358	1	0	1	2
ST14	1	0	0	1
ST42	0	0	1	1
ST71	0	0	1	1
ST96	1	0	0	1
ST292	1	0	0	1
ST297	1	0	0	1
ST314	0	0	1	1
ST321	0	1	0	1
ST365	0	0	1	1
ST413	1	0	0	1
ST544	1	0	0	1
ST592	1	0	0	1
ST1543	1	0	0	1
ST1561	1	0	0	1
ST1610	1	0	0	1
ST1628	0	1	0	1
ST1651	1	0	0	1
ST8333	0	1	0	1
Novel	1	1	0	2

### Antimicrobial Resistance and Virulence Gene Prediction

**FIGURE 4:**
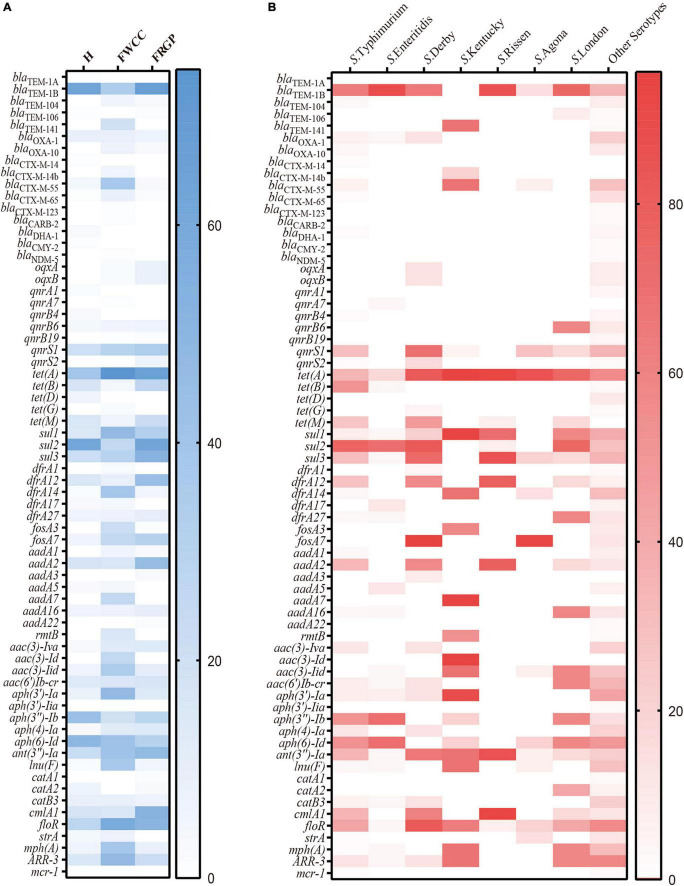
Heatmap of antimicrobial resistance genes according to sampling sources: **(A)** serotypes; **(B)** H, human; FWCC, frozen whole chicken carcasses; FGRP, frozen raw ground pork.

Overall, up to 68 antimicrobial-resistant (AMR) genes were identified among 192 (88.9%, 192/216) *Salmonella* isolates, which belonged to 11 different antibiotic resistance categories, with a high prevalence of *bla*_TEM–1B_ encoding resistance to penicillin, *bla*_CTX–M–55_ encoding resistance to third-generation cephalosporin, *qnrS1* encoding resistance to fluoroquinolone, *tet(A)* encoding resistance to tetracycline, *sul2* encoding resistance to SUL, *aph(6)-Id* encoding resistance to streptomycin, and *mph(A)* encoding resistance to AZI. Moreover, *Salmonella* isolates cultured from livestock meat harbor more AMR genes than those obtained from diarrhea patients (Figure 4A). Regarding the distribution of AMR genes in different serotypes, it appears that *S*. Typhimurium and its monophasic variant hold broader diversity (Figure 4B).

**FIGURE 5:**
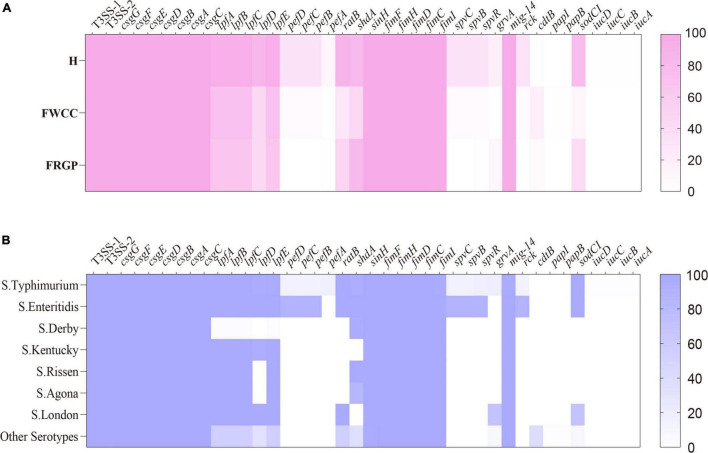
Heatmap of antimicrobial virulence genes according to sampling sources: **(A)** serotypes; **(B)** H, human; FWCC, frozen whole chicken carcasses; FGRP, frozen raw ground pork.

After screening the genome data of 216 *Salmonella* isolates, we found 120 potential virulence genes (Figure 5). The typical virulence factors carried on *Salmonella* Pathogenicity Island 1 and 2 (SPI-1 and SPI-2) were detected in all the examined isolates. The chromosome-encoded type III secretion system 1 (T3SS-1), type III secretion system 2 (T3SS-2), and fimbrial adherence genes were common to all strains. The plasmid-encoded fimbrial genes *pefBCD* and plasmid-mediated genes *spvR*, *spvB*, *spvC*, and *rck* were detectable more often (33.3%–34.5%) in isolates cultured from diarrhea patients than isolates cultured from frozen whole chicken carcasses with low proportion (5.7%). It is worth noting that plasmid-mediated genes *pefBCD*, *spvR*, *spvB*, *spvC*, and *rck* which have been mentioned earlier were detected solely within *S*. Typhimurium and its monophasic variant and *S*. Enteritidis. Thus, it seems that the common serotypes in human infections carried more virulence genes. In addition, 8.8% (19/216) isolates harbor the gene *cdtB* encoding typhoid toxins, and the following serotypes: *S*. Indiana, *S*. Muenster, *S.* Schwarzengrund, *S*. Give, and *S*. Goldcoast. Of these, except for one isolate cultured from a diarrhea patient, rest were cultured from livestock meat. Therefore, *cdtB* was also detected in more serotypes other than *S*. Typhi.

### Detection of Third-Generation Cephalosporin Resistance Mechanisms in Third-Generation Cephalosporin Resistance Isolates

**FIGURE 6:**
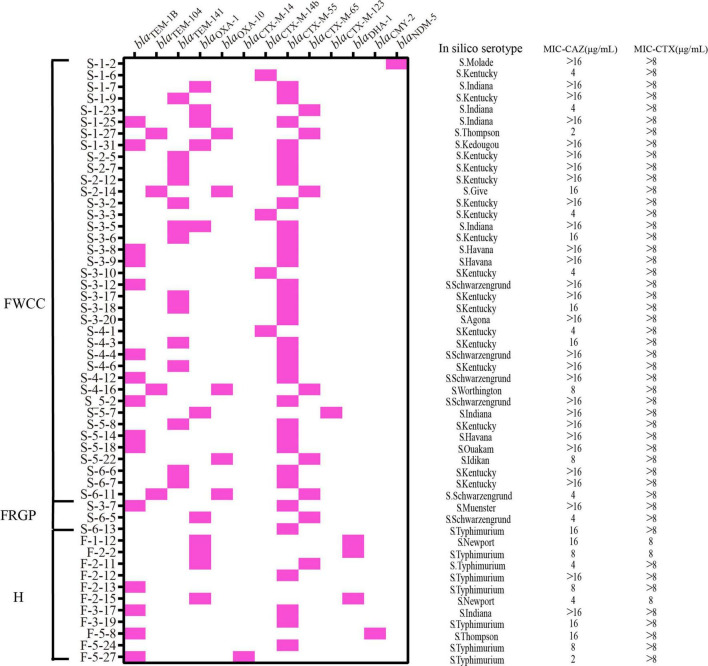
Distribution of extended-spectrum β-lactamase (ESBL), AmpC and Carbapenemase β-lactamase genes, serotypes, minimal inhibitory concentration (MIC) of ceftazidime (CAZ), and MIC of cefotaxime (CTX) in the third-generation cephalosporin-resistant *Salmonella* isolates from different sources (*n* = 52). H, human; FWCC, frozen whole chicken carcasses; FGRP, frozen raw ground pork.

The prevalence of third-generation cephalosporin resistance *Salmonella* isolates was 24.1% (52/216). Of these, 38 (73.1%, 38/52) were isolates collected from frozen whole chicken carcasses, 11 (21.2%, 11/52) from patients with diarrhea, and only 3 from raw ground pork (Figure 6). Furthermore, the genomic analysis of AMR genes indicated that the reason for these isolates of third-generation cephalosporin resistance was producing extended-spectrum β-lactamases (ESBLs). Subsequently, the results show that the most prevalent gene was *bla*_CTX–M–55_ (61.5%, 32/52), with *S*. Kentucky being the dominant serotype, followed by *bla*_OXA–1_ (21.2%, 11/52) and *bla*_CTX–M–65_ (15.4%, 8/52). In particular, the NDM-5-producing *S*. Molade ST544 strain was recovered from whole chicken carcasses, and the *bla*_NDM–5_ gene was harbored by a 46,161-bp IncX3 plasmid. The *bla*_NDM–5_-bearing plasmid shared 100% query coverage and sequence identity with the plasmid, pNDM5_SH160, isolated from pork in China (Gao et al., 2020). The IncX3 plasmid contains a composite cassette, consisting of *ISSwil*-*IS3000*-Δ*ISAba125*-*IS5*-*bla*_NDM–5_-*ble*_MBL_-*trpF*-*dsbC*-*IS26*-*ctuA1*-Δ*umuD*. In addition, two acquired antimicrobial resistance genes, *aac(6’)-Iaa* and *fosA7*, were carried on the chromosomal genes.

### Detection of Ciprofloxacin Resistance Mechanisms in Ciprofloxacin-Resistant Isolates

**FIGURE 7:**
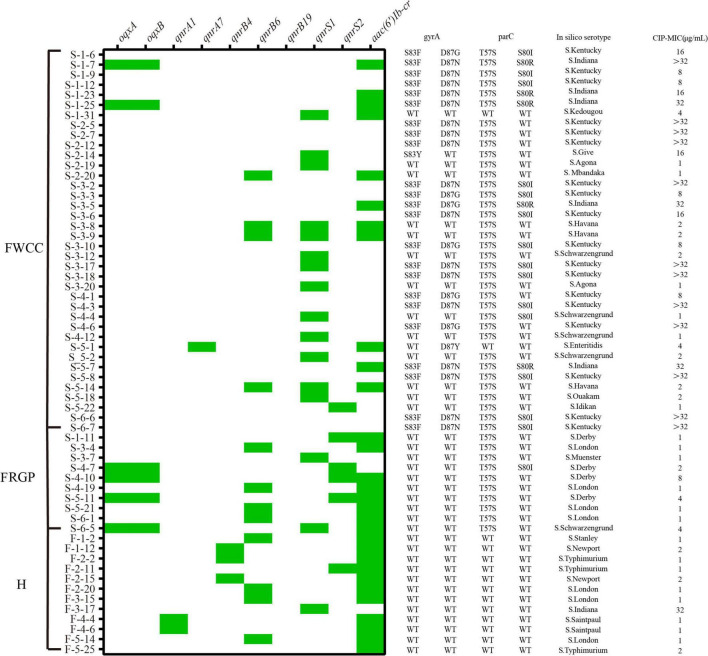
Distribution of plasmid-mediated quinolone resistance (PMQR) genes, quinolone resistance-determining regions (QRDRs) mutations, serotypes, minimal inhibitory concentration (MIC) of ciprofloxacin (CIP) in the ciprofloxacin-resistant isolates from different sources (*n* = 60). H, human; FWCC, frozen whole chicken carcasses; FGRP, frozen raw ground pork.

Sixty out of 216 *Salmonella* were resistant to CIP (27.8%, 60/216) (Figure 7). The quinolone resistance-determining regions (QRDRs) mutations and plasmid-mediated quinolone resistance (PMQR) genes have been screened for quinolone resistance determinants based on the genome sequence. In total, five QRDRs point mutations were identified: two in *gyrA* (S83F, S83Y) and three in *parC* (T57S, S80I, S80R), respectively. No *gyrB* and *parE* mutations were found. Consequently, 10 types of QRDR gene mutation patterns were observed (Table 5). None of the CIP-resistant isolates from diarrheal patients and only one from frozen whole chicken carcasses found mutation in the QRDRs. The proportion of PMQR genes was as follow: *aac(6’)Ib-cr* (50.0%, 30/60), *qnrS1* (26.7%, 16/60), *qnrB6* (20.0%, 12/60), *qnrS2* (10.0%, 6/60), *oqxAB* (10.0%, 6/10), *qnrB4* (5.0%, 3/60), *qnrA1* (3.3%, 2/60), and *qnrA7* (1.7%, 1/60). In addition, 13.3% (8/60) of the CIP-resistant isolates combined both resistance mechanisms, including missense mutations in the QRDRs and PMQR genes, showing a relatively high resistance level against CIP (MIC ≥ 16 μg/mL).

**TABLE 5:** Mutations of *gyrA*, *gyrB*, *parC*, and *parE* genes in ciprofloxacin-resistant *salmonella* (*n* = 60).

QRDR mutation						No. isolate

*gyrA*		*gyrB*	*parC*		*parE*	
WT	WT	WT	T57S	WT	WT	20
WT	WT	WT	WT	WT	WT	13
S83F	D87N	WT	T57S	S80I	WT	10
S83F	D87N	WT	T57S	S80R	WT	4
S83F	D87G	WT	T57S	S80I	WT	3
S83F	D87N	WT	T57S	WT	WT	3
WT	WT	WT	T57S	S80I	WT	2
S83F	D87G	WT	T57S	WT	WT	2
S83F	D87G	WT	T57S	S80R	WT	1
S83Y	WT	WT	T57S	WT	WT	1
WT	D87Y	WT	WT	WT	WT	1

*WT, wild type.*

## Discussion

Previous studies have shown that the prevalence rates of *Salmonella* in livestock meat were 9.5% (23/242) (Chen et al., 2019), 42.6% (245/530) (Chen et al., 2021), 63.6% (302/475) (Zhang et al., 2018), 42.1% (224/532) (Rortana et al., 2021), 3.1% (31/850) (Da Cunha-Neto et al., 2018), 29.53% (269/911) (Xu et al., 2020), and 29.33% (105/358) (Ma et al., 2017), which are slightly different from this study. This discrepancy might be related to seasons, sample size, collection and processing, and regional differences. In addition, the lower detection rate in diarrhea patients was likely due to salmonellosis being a self-limited illness. Our research objects do not cover all populations, only focusing on patients with diarrhea.

Serotyping is one of the gold standard bacterial typing methods. From the perspective of human disease, the *Salmonella* spp. is traditionally divided into zoonotic *Salmonella* serotypes and human-adapted *Salmonella* serotypes. In this study, *S*. Typhimurium and its monophasic variant and *S*. Enteritidis were the most common serotypes found among patients with diarrhea, consistent with some studies in other areas of China (Zhan et al., 2017; Liu et al., 2021; Wu L. J. et al., 2021). However, *S*. Infantis and *S*. Enteritidis were the most common disease-causing serotypes in the EU (European Food Safety Authority, 2021a). *S*. Typhimurium and its monophasic variant, *S*. Derby, and *S*. Kentucky were the most dominant serotypes in livestock meat samples, and *S*. Kentucky was most detected in frozen whole chicken carcasses, which may result from its metabolic adaptation to the chicken cecum (Cheng et al., 2015). Therefore, livestock meat can be a vector of human salmonellosis.

This study used traditional and *in silico* serotyping based on WGS to determine the serotype. Results demonstrated 93.52% concordance between the two methods, close to Cooper et al. (2020) (89.2%) and Lyu et al. (2021) (96.5%). Several untypable and/or ambiguous serotypes were addressed by *in silico* serotyping. This result suggests that *in silico* serotyping is more accurate and reliable. Thus, we mainly adopted the results of the *in silico* serotyping in the discussion. Although traditional serotyping may have certain limitations, this approach is widely adopted by most clinical laboratories in China due to its operability and low cost.

Our results showed that the resistance of frozen whole chicken carcasses to the highest priority “critically important antimicrobials” CAZ, CTX, and CIP was significantly higher than other samples. Thus, once people are infected with resistant *Salmonella* from livestock meat, the difficulty of treatment would increase. In addition, the proportion of MDR isolates from different sources was high, in agreement with the range from 77.1% to 85.90% (Zhang et al., 2018; Chen et al., 2021; Wu B. et al., 2021) in other areas of China, and the 87.2% detected by Kim et al. (2012) in South Korea. The prevalence of whole chicken carcasses isolates with R-type ACSSuT was remarkably higher than this from the diarrhea patient samples. Previous studies have reported that the mortality rate of *Salmonella* infection with R-type ACSSuT was 4.8-fold higher than the general population (Angulo and Molbak, 2005). The management and control the use of antibiotics in food animals is crucial.

The presence of AMR genes could be responsible for phenotypic resistances. In this study, 213 *Salmonella* isolates were resistant to at least one antibiotic. However, resistance genes were only screened among 192 *Salmonella* isolates, which suggests that antimicrobial resistance was not only associated with the presence/absence of resistance genes but also with other mechanisms that could also affect this phenomenon. Virulence genes have a significant influence on *Salmonella* infection. In this study, the *spvR*, *spvB*, *spvC*, *pefBCD*, and *rck* located on the virulence plasmid of *Salmonella* were detected mainly in isolates cultured from diarrhea patients. Still, those genes were also detected in frozen whole chicken carcasses. In addition, these virulence factors were particularly associated with the *S*. Typhimurium and its monophasic variant and *S*. Enteritidis, and none of these genes were detected in the other serotypes. It has been demonstrated that non-typhoid bacteremia was closely associated with *spv* locus (Guiney and Fierer, 2011), *pef* is responsible for resistance to complement killing and adhesion to intestinal cells (Rychlik et al., 2006), and *rck* plays a key role in the invasion of different host cells (Mambu et al., 2017). Remarkably, the *spv*, *pef*, and rck genes detected exclusively within *S*. Enteritidis were correlated with high *in vitro* infectious livestock-borne strains (Kuijpers et al., 2019). Thus, livestock meat may be an underlying infection source of salmonellosis. Notably, in our study, virulence gene screening-detected *cdtB* gene encoding typhoid toxins in 19 non-typhoid *Salmonella* strains. Past studies have shown that *cdtB* positive isolates may be more likely to cause invasive disease (Rodriguez-Rivera et al., 2015). Particularly, *cdtB* positive isolates were primarily found in livestock meat, which may burden the public healthcare system.

*Salmonella* infection is more common in infants and the elderly, and immunocompromised patients. However, the use of fluoroquinolones in children is limited due to the potential side effects of these agents (Schaad, 2005), which makes third-generation cephalosporin a prime candidate for salmonellosis therapeutics. Special attention needs to be paid to the treatment of salmonellosis in this age group. In this study, we identified ESBLs genes as the most important determinants of third-generation cephalosporin resistance *Salmonella*, *bla*_CTX–M–55_ was the predominant ESBL gene and was mainly detected from frozen whole chicken carcasses, and the result was consistent with other studies (Nadimpalli et al., 2019; Zhang et al., 2019), specifically, the *bla*_CTX–M–55_ was predominantly detected among the *S*. Kentucky. However, *bla*_CTX–M–1_, *bla*_CTX–M–14_, and *bla*_CTX–M–65_ were frequently reported from animals, especially livestock in other studies (Rodríguez et al., 2009; Eller et al., 2013; Pietsch et al., 2021). In addition, AmpC β-lactamases gene *bla*_DHA_ and *bla*_CMY–2_, and carbapenemase gene *bla*_NDM–5_ were also found. When ESBL-producing *Salmonella* from livestock meat is transmitted to children through the fecal-oral route, the treatment may become an intractable problem.

Mutations in the QRDRs of the chromosomal and carrying the PMQR gene are the principal mechanisms against quinolones (Cuypers et al., 2018). In this study, 28.33% of CIP-resistance isolates only harbored mutations within the QRDRs, which showed that the resistance to CIP was high (MIC ≥ 8 μg/mL). This revealed that mutations in the QRDRs may lead to high-level CIP resistance. Interestingly, the CIP*-*resistance isolates from diarrhea patients did not harbor any missense mutations in the QRDRs. The reasons for CIP-resistance will be explained further. Among the CIP-resistance isolates, the most common PMQR gene was *aac(6*′*)-Ib-cr*. These observations differed from the previous studies, which determined *qnr* was the most prevalent PMQR gene (Wasyl et al., 2014; Soares et al., 2019; Herrera-Sánchez et al., 2021), and the CIP*-*resistance isolates from diarrhea patients carried at least one PMQR gene.

Finally, serovar-specific cgMLST can be used for reasonable, reproducible, and reliable high-resolution classification of *Salmonella* WGS data (Dangel et al., 2019). In this study, to assess the possibility of the transmission of *Salmonella* from different sources, we evaluated the number of allelic differences to determine relatedness based on cgMLST. It is not surprising that no isolates from various sources have the same allelic completely due to exhibited spatial and temporal differences. However, there was an association between strains of different sources based on allelic difference cutoffs of ≤ 7 allelic differences. The above result indicates a strong relationship between the *Salmonella* isolates obtained from patients and livestock meat. Thus, there is a need to take corresponding preventive measures to reduce *Salmonella* transmission through the food chain.

## Conclusion

In conclusion, due to the temporal and spatial changes and host adaptability, the prevalence of *Salmonella* has both diversity and a close relationship with different hosts. Livestock meat *Salmonella* isolates have more antimicrobial resistance than isolates obtained from diarrheal patients and could be an important source of human *salmonella* infection, presenting a potential public health risk.

## Data Availability Statement

The datasets presented in this study can be found in online repositories. The names of the repository/repositories and accession number(s) can be found below: https://www.ncbi.nlm.nih.gov/, PRJNA809954.

## Author Contributions

RW and YG contributed to conceptualization and writing, reviewing, and editing the manuscript. RW and WZ contributed to methodology and wrote the original draft. RW contributed to software, formal analysis, data curation, and visualization. RW, WZ, and YG contributed to validation. XH, YZ, and JT contributed to investigation. RW, WZ, XH, HW, JT, MD, and MZ contributed to resources. WZ contributed to project administration. YG contributed to funding acquisition and supervision. All authors have read and agreed to the published version of the manuscript.

## Conflict of Interest

The authors declare that the research was conducted in the absence of any commercial or financial relationships that could be construed as a potential conflict of interest.

## Publisher’s Note

All claims expressed in this article are solely those of the authors and do not necessarily represent those of their affiliated organizations, or those of the publisher, the editors and the reviewers. Any product that may be evaluated in this article, or claim that may be made by its manufacturer, is not guaranteed or endorsed by the publisher.

## Footnotes

1
http://www.genomicepidemiology.org/

2
https://lfz.corefacility.ca/sistr-app/

3
http://www.mgc.ac.cn/cgi-bin/VFs/
