# Corrigendum: Whole-genome sequencing provides insight into antimicrobial resistance and molecular characteristics of *Salmonella* from livestock meat and diarrhea patient in Hanzhong, China

**DOI:** 10.3389/fmicb.2022.981414

**Published:** 2022-08-02

**Authors:** Rui Weng, Yihai Gu, Wei Zhang, Xuan Hou, Hui Wang, Junqi Tao, Minghui Deng, Mengrong Zhou, Yifei Zhao

**Affiliations:** ^1^Department of Microbiology, 3201 Hospital, School of Medicine, Xi'an Jiaotong University, Hanzhong, China; ^2^Department of Medical Technology, Shaanxi University of Chinese Medicine, Xianyang, China; ^3^The Second Clinical Medical College of Nanchang University, Nanchang, China

**Keywords:** *Salmonella*, antimicrobial resistance, whole-genome sequencing, livestock meat, diarrhea patients

In the published article, there was an error in affiliation (1). Instead of “Department of Microbiology, School of Medicine, Xi'an Jiaotong University, Hanzhong, China,” it should be “Department of Microbiology, 3201 Hospital, School of Medicine, Xi'an Jiaotong University, Hanzhong, China.”

The authors apologize for this error and state that this does not change the scientific conclusions of the article in any way. The original article has been updated.

## Publisher's note

All claims expressed in this article are solely those of the authors and do not necessarily represent those of their affiliated organizations, or those of the publisher, the editors and the reviewers. Any product that may be evaluated in this article, or claim that may be made by its manufacturer, is not guaranteed or endorsed by the publisher.

